# CD100/Sema4D Increases Macrophage Infection by *Leishmania (Leishmania) amazonensis* in a CD72 Dependent Manner

**DOI:** 10.3389/fmicb.2018.01177

**Published:** 2018-06-05

**Authors:** Mariana K. Galuppo, Eloiza de Rezende, Fabio L. Forti, Mauro Cortez, Mario C. Cruz, Andre A. Teixeira, Ricardo J. Giordano, Beatriz S. Stolf

**Affiliations:** ^1^Department of Parasitology, Institute of Biomedical Sciences, University of São Paulo, São Paulo, Brazil; ^2^Department of Biochemistry, Institute of Chemistry, University of São Paulo, São Paulo, Brazil; ^3^Department of Immunology, Institute of Biomedical Sciences, University of São Paulo, São Paulo, Brazil

**Keywords:** CD100, *Leishmania amazonensis*, macrophages, phagocytosis, infection index, CD72

## Abstract

Leishmaniasis is caused by trypanosomatid protozoa of the genus *Leishmania*, which infect preferentially macrophages. The disease affects 12 million people worldwide, who may present cutaneous, mucocutaneous or visceral forms. Several factors influence the form and severity of the disease, and the main ones are the *Leishmania* species and the host immune response. CD100 is a membrane bound protein that can also be shed. It was first identified in T lymphocytes and latter shown to be induced in macrophages by inflammatory stimuli. The soluble CD100 (sCD100) reduces migration and expression of inflammatory cytokines in human monocytes and dendritic cells, as well as the intake of oxidized low-density lipoprotein (oxLDL) by human macrophages. Considering the importance of macrophages in *Leishmania* infection and the potential role of sCD100 in the modulation of macrophage phagocytosis and activation, we analyzed the expression and distribution of CD100 in murine macrophages and the effects of sCD100 on macrophage infection by *Leishmania (Leishmania) amazonensis.* Here we show that CD100 expression in murine macrophages increases after infection with *Leishmania*. sCD100 augments infection and phagocytosis of *Leishmania (L.) amazonensis* promastigotes by macrophages, an effect dependent on macrophage CD72 receptor. Besides, sCD100 enhances phagocytosis of zymosan particles and infection by *Trypanosoma cruzi*.

## Introduction

Leishmaniasis is a complex of diseases caused by trypanosomatid protozoa of the genus *Leishmania*, which can be grouped into cutaneous or visceral forms (Alvar et al., 2012). Due to their considerable impact on global health, leishmaniasis are listed among the priority endemic diseases of the World Health Organization (WHO). It is estimated that 12 million people in the world are infected, and about 1.5 million new cases are reported every year (Alvar et al., 2012). The disease currently affects 98 countries, and in Brazil it has been observed an increase in the number of cases in recent years, accompanied by their geographical spread (WHO, 2015). Many species of *Leishmania* cause leishmaniasis in humans. The parasite species as well as the host immune response are the main determinants of the clinical form and the course of the disease (McMahon-Pratt and Alexander, 2004). The available treatment options for many *Leishmania* species and clinical forms are toxic and not always effective (McGwire and Satoskar, 2014). Thus, understanding the molecular basis of infection may be an important step toward the development of novel therapeutic approaches for this disease.

*Leishmania* is an intracellular parasite that infects mononuclear phagocytic cells of vertebrates. Macrophages are the main parasite host cells and their activation is crucial for the resolution of the infection (Iniesta et al., 2002, 2005). The general process of phagocytosis is an essential mechanism of the innate immune response by which phagocytes such as macrophages internalize microorganisms, dead or dying cells, and debris. It is an actin-dependent process triggered by the interaction between phagocyte’s receptors and ligands of the particle to be engulfed (May and Machesky, 2001; Underhill and Goodridge, 2012).

The receptors most frequently involved in the phagocytosis of *Leishmania* are complement receptors 3 (CR3) and 1 (CR1), mannose receptor (MR), fibronectin receptor (FnR) and receptors Fc gamma (FcγRs) (Blackwell et al., 1985; Mosser and Edelson, 1985; Wyler et al., 1985; Da Silva et al., 1989; Guy and Belosevic, 1993). The receptors and internalization pathways may vary depending on the parasite stage (Ueno and Wilson, 2012). The actin cytoskeleton also plays important role in *Leishmania* binding and internalization, and was studied in more detail in *L. (Leishmania) donovani* (May et al., 2000; Roy et al., 2014; Podinovskaia and Descoteaux, 2015). The association of polymerized F-actin and parasite binding was also shown for *L. (Viannia) braziliensis* (Azevedo et al., 2012), *L.* (*L.*) *amazonensis*, and *L. (Leishmania) major* (Courret et al., 2002).

CD100, also known as Sema4D, belongs to class IV of semaphorins and was the first semaphorin described in the immune system (Bougeret et al., 1992; Mizui et al., 2009; Ch’ng and Kumanogoh, 2010). It exists as a membrane bound dimer or as a soluble protein originated by proteolytic cleavage (Elhabazi et al., 2003; Basile et al., 2007) that interacts with specific receptors, mainly plexin B1 (Basile et al., 2004; Conrotto et al., 2005; Nkyimbeng-Takwi and Chapoval, 2011) and CD72 (Kumanogoh et al., 2000; Ishida et al., 2003; Smith et al., 2011).

CD100 is expressed by the majority of the cells of the hematopoietic system, including B and T lymphocytes, natural killer and myeloid cells, and its expression usually increases upon activation (Elhabazi et al., 2003). Membrane CD100 is cleaved from the cell surface in an activation-dependent manner (Kikutani and Kumanogoh, 2003). In fact, sCD100 is shed by activated T and B cells, and sCD100 can be detected in sera of mice immunized with T-cell–dependent antigens or in sera of MRL/lpr mice with autoimmune disease (Wang et al., 2001). In mice, sCD100 increases proliferation, differentiation and IgG1 production by stimulated B cells (Kumanogoh et al., 2000; Shi et al., 2000). CD100 mediates DC-T cell interaction increasing activation, proliferation and differentiation of T cells (Shi et al., 2000; Kumanogoh et al., 2002; Mizui et al., 2009), and inducing DC maturation (Kumanogoh et al., 2002). In humans, sCD100 inhibits migration of B cells (Delaire et al., 2001), monocytes and immature DCs (Chabbert-de Ponnat et al., 2005). It also increases IL-10 secretion and reduces IL-6, IL-8 and TNF-α in monocytes and DCs (Chabbert-de Ponnat et al., 2005).

Although it is known that CD100 is expressed in macrophages (Kikutani and Kumanogoh, 2003; Nkyimbeng-Takwi and Chapoval, 2011), few studies have reported its effects on these cells. One of them analyzed the role of macrophage shed sCD100 in tumor angiogenesis (Sierra et al., 2008). Other showed that CD100 is also important in glomerular nephritis, enhancing T and B cell activation and the recruitment of macrophages (Li et al., 2009). We have shown that macrophages from human atherosclerotic plaques express CD100, and that sCD100 inhibits internalization of oxidized LDL (Luque et al., 2013). We have also shown that CD100 participates on the interaction between human monocyte and endothelial cell by binding to plexins B1 and B2 (Luque et al., 2015).

The effect of sCD100 on oxLDL phagocytosis by macrophage, the main *Leishmania* host cells, prompted us to study the expression of this molecule and its effects on the phagocytosis of this parasite. *Leishmania* lesions are characterized by intense inflammatory infiltrates, and thus sCD100 is probably shed by activated T and B cells near infected and non-infected macrophages. Here we show that sCD100 increases macrophage phagocytosis of *L.* (*L*.) *amazonensis* promastigotes, *Trypanosoma cruzi* trypomastigotes and zymosan particles. In addition, we demonstrated that sCD100 effects depend on macrophage CD72, a receptor for CD100. This is the first report of CD100 effect on a parasitic infection, and further studies should address the role of this molecule in animal models of leishmaniasis.

## Materials and Methods

### *Leishmania* (*Leishmania*) *amazonensis* Promastigotes

Promastigotes of *L*. (*L*.) *amazonensis* LV79 strain (MPRO/BR/72/M1841) were cultured at 24°C in M199 medium supplemented with 10% fetal calf serum (FCS). Parasites were subcultured every 7 days at an initial inoculum of 2 × 10^6^/mL.

When indicated, promastigotes were incubated for 2 h with 200 ng/ml sCD100 in M199 at 24°C, centrifuged at 2,500 × *g* for 10 min and resuspended in RPMI 1640 medium supplemented with 10% FBS for subsequent infection of peritoneal macrophages.

### Ethics Statement

All animals were used according to the Brazilian College of Animal Experimentation guidelines, and the protocols were approved by the Institutional Animal Care and Use Committee (CEUA) of the University of São Paulo (protocol number 001/2009).

### Recombinant sCD100 Production

sCD100 protein fused to Fc portion of IgG was produced in HEK (human embryonic kidney) 293T cells transfected with CD100-Fc plasmid, kindly given by Kumanogoh et al. (2000). Briefly, 7,5 × 10^6^ HEK cells were plated in DMEM with 5% serum “low-IgG” (Life Technologies) supplemented with 2 mM L-glutamine, 1 mM sodium pyruvate and 1× antibiotic-antimycotic solution (Life Technologies). Ten micrograms of CD100-Fc plasmid were mixed with 1 mL of a 150 mM NaCl solution and then with 100 μL of a polyethylenimine solution (PEI, Sigma) at 0.45 mg/mL. The total volume was added slowly to each dish, which was then incubated at 37°C with 5% CO2 for 7 days. After this period, supernatants of cell cultures were collected, filtered through a 0.22 μm membrane and centrifuged at 7,500 × *g* for 10 min. PMSF to 100 μM was added to the supernatant and proteins were precipitated with 60% w/v ammonium sulfate under slow stirring at 4°C for 24 h. Two successive centrifugations at 10,000 × *g* for 45 min were performed, precipitates were resuspended in PBS and centrifuged. The supernatant was incubated with protein G beads (1 mL Protein G Sepharose 4FF GE beads/50 mL supernatant) under rotation at 4°C for 24 h. The suspension was then centrifuged at 800 × *g* for 5 min and the beads were transferred to a chromatography column (Bio-Rad). The column was washed twice with 5 mL ice cold PBS and protein was eluted in aliquots of 500 μL using 0.1 M glycine buffer, pH 3.0, neutralized by 50 μl 1 M Tris buffer pH 8.0. Protein concentrations were determined by Bradford (Bio-Rad) and sCD100 was analyzed by SDS–PAGE and Western blot.

### Macrophage Infection With *Leishmania* and Phagocytosis of Zymosan

Peritoneal macrophages were isolated as previously described (Velasquez et al., 2016). We then transferred 8 × 10^5^ cells in RPMI 1640 pH 7.2 to each well of 24 well plates laid with 13 mm circular coverslips. After 2 h of incubation at 37°C in 5% CO_2_, the medium was changed to RPMI with 10% FCS with or without 100 or 200 ng/mL sCD100 and cells were incubated until the next day. Infection was performed with *L. (L.) amazonensis* promastigotes at the beginning of stationary phase (day four) using a multiplicity of infection (MOI) of 5:1 for 4 h. After removing the non-internalized parasites, cells were further incubated for 24, 48, and 72 h with or without (control) sCD100. Control experiments for the effect of sCD100 Fc portion on *Leishmania* infection were performed using recombinant IgG1 and FcR blocker, and are described latter. Zymosan particles were incubated with macrophages at a ratio of 1:1 for 1 h to analyze the phagocytosis index. In *Leishmania* and zymosan experiments cells were fixed with methanol, stained with Giemsa and mounted with Entellan (Merck). One hundred macrophages were analyzed per glass slide to determine the proportion of infected cells (IM), the total number of amastigotes (AMA), amastigotes/infected macrophage and Infection Index or Phagocytosis Index (II = IM x AMA). Three coverslips were prepared for each condition.

### Cell Infection by *Trypanosoma cruzi*

Experimental procedures were carried as previously described (Teixeira A. A. et al., 2015). Briefly, peritoneal macrophages were seeded in 24 well plates laid with 13 mm circular coverslips as described above, with or without 200 ng/mL sCD100. Cells were then infected with tissue culture-derived trypomastigotes (Y strain) at MOI = 10 with or without 200 ng/mL CD100 (*N* = 3 for each condition) for 2 h at 37°C. The cells were then washed 10 times with PBS and further cultivated for 24 h to allow parasite differentiation. Macrophages were fixed with 4% paraformaldehyde, stained with anti-*T. cruzi* polyclonal antibody and propidium iodide and photographed with an epifluorescence microscope. Quantification was performed by counting the number of total and infected cells in at least 4 different fields (20 × magnification) for each replicate.

### Macrophage Infections With sCD100 and Fc Receptor Blocker, Human Recombinant IgG1, or Anti-CD72

To monitor the effect of sCD100 Fc portion on *Leishmania* infection, incubations with *L. (L.) amazonensis* were performed in the presence of sCD100 and FcR blocker, and in the presence of human recombinant Fc portion of IgG1. To evaluate the role of CD72 in sCD100 effects, infections were done in the presence of anti-CD72. For FcR blocking: macrophages were plated and incubated with Fc receptor blockers (CD16 and CD32- BD Biosciences) at 0.1 ug/mL for 10 min, and then 200 ng/mL of sCD100 was added. *Leishmania* was added in the following day in the presence of FcR blocker and sCD100 for 4 and 24 h. For IgG1, macrophages were plated and incubated with 70 ng/mL of human recombinant IgG1 Fc (R&D Systems) and 200 ng/mL sCD100, and infected in the following day in the presence of the same proteins. For anti-CD72, plated macrophages were incubated with 10 μg/mL of anti-CD72 H-96 (Santa Cruz Biotechnology) and 1 h later sCD100 was added to 200 ng/mL. Infection was performed in the following day in the presence of both proteins. Control conditions included incubation only with sCD100 and with each treatment (Fc blocker or Fc-IgG1) separately, as well as untreated infected macrophages. The analysis and comparisons were based on infection rates.

### Immunofluorescence for Phagocytosis and for F-Actin, CD100, and CD72 Labeling

Resident peritoneal macrophages from BALB/c mice were plated on glass slides as described and incubated with no stimulus or with 100 or 200 ng/mL of sCD100, or 100 or 200 ng/mL of BSA overnight.

For phagocytosis and for F-actin and CD100 labeling: Promastigotes of *L*. (*L*.) *amazonensis* were added in the proportion of 10 parasites per cell in the presence or absence of sCD100 or BSA and the plate was kept on ice for 2 h, and at 33°C with 5% CO_2_ for different periods. After washing, macrophages were fixed with 4% paraformaldehyde for 10 min, washed with PBS 1X with 2% FBS, incubated for 30 min in 50 mM ammonium chloride and washed in PBS 1X with 2% FBS.

For labeling of CD100 and actin, cells were permeabilized with TBS containing 1% BSA and 0.1% Triton for 10 min. Slides were washed and incubated overnight with anti-CD100 (eBioscience) at 1:50 dilution, and after washing were incubated with anti-Rat IgG Alexa Fluor 568 (Thermo Scientific) diluted 1: 1000 and DAPI 1: 600 for 1 h. Alternatively, cells were incubated with phalloidin Texas Red (Molecular Probes) 1: 500 and DAPI 1: 600 for 1 h. In both cases, coverslips were washed five times with PBS and mounted in ProLong (Molecular Probes).

For analysis of phagocytosis glass slides were incubated overnight with anti-*Leishmania* serum diluted 1:75 in PBS 1X, washed five times with PBS and incubated for 1 h with a mix containing anti-mouse IgG Alexa Fluor 488 (Thermo Scientific) 1: 1000. After washing, permeabilization with TBS containing 1% BSA and 0.1% Triton for 10 min and further washing, phalloidin Texas Red (Molecular Probes) 1: 500 and DAPI 1: 600 were added for 1 h. After five washes, cells were mounted in ProLong (Molecular Probes). For calculation of the phagocytosis index, 500 macrophages were analyzed and promastigotes were quantified as attached (labeled in green and blue) or internalized (labeled only with blue- DAPI).

For CD72 labeling, slides containing non-infected macrophages were fixed as described. After washing they were incubated with anti-CD72 antibody M-96 (Santa Cruz Biotechnologies) 1:75 overnight, washed and incubated for 2 h with DAPI at 1: 600 and anti-rabbit Alexa Fluor 488 (Thermo Scientific) 1: 100. After labeling, coverslips were washed three times in PBS 1X with 2% FBS and cells were mounted in ProLong (Molecular Probes).

For CD100 quantification, images were acquired in a DMI6000B/AF6000 (Leica) fluorescence microscope coupled to a digital camera system (DFC 365 FX) and analyzed with the Image J program.

For actin and CD72 labeling and for the phagocytosis assay, images were captured using a Zeiss LSM 780 confocal laser scanning inverted microscope (Carl Zeiss, Germany) in a 1024 × 1024 pixel format. Image stacks comprised 8 images captured with a Plan-Apochromat 63×/1.4 DIC Oil M27 objective (Zeiss), applying a zoom factor of 1.0. Step intervals along the Z-axis ranged from 450 nm. Image acquisition and processing were performed using the Zen 2011 software (Zeiss, version 11.00.190).

### SDS–PAGE and Western Blot

SDS–PAGE (running gels with 10% acrylamide: bisacrylamide) and Western blots were performed as we previously described (Teixeira P. C. et al., 2015), using the following antibodies and incubation conditions: anti β-actin (Imuny, Brazil) 1: 1000 overnight and anti-rabbit IgG (H + L) (Imuny, Brazil) 1: 2000 for 1 h; anti-CD72 H-96 (Santa Cruz Biotechnology) 1: 200 overnight and anti-rabbit HRP (Imuny) 1: 1000 for 1 h, anti-GAPDH (Sigma-Aldrich) 1: 10000 overnight and anti-rabbit HRP (Imuny) 1: 1000 for 1 h. Normalizations for CD72 were done using anti-GAPDH while actin polymerization was estimated by F/G ratio.

### Statistical Analysis

Statistical analyses were done using *t*-test or one-way ANOVA followed by Tukey’s multiple comparison test, depending on the number of samples. Data were considered statistically different (^∗^) when *p* < 0.05.

## Results

### CD100 Expression Increases in Macrophages During Infection With *Leishmania* (*L*.) *amazonensis*

**FIGURE 1:**
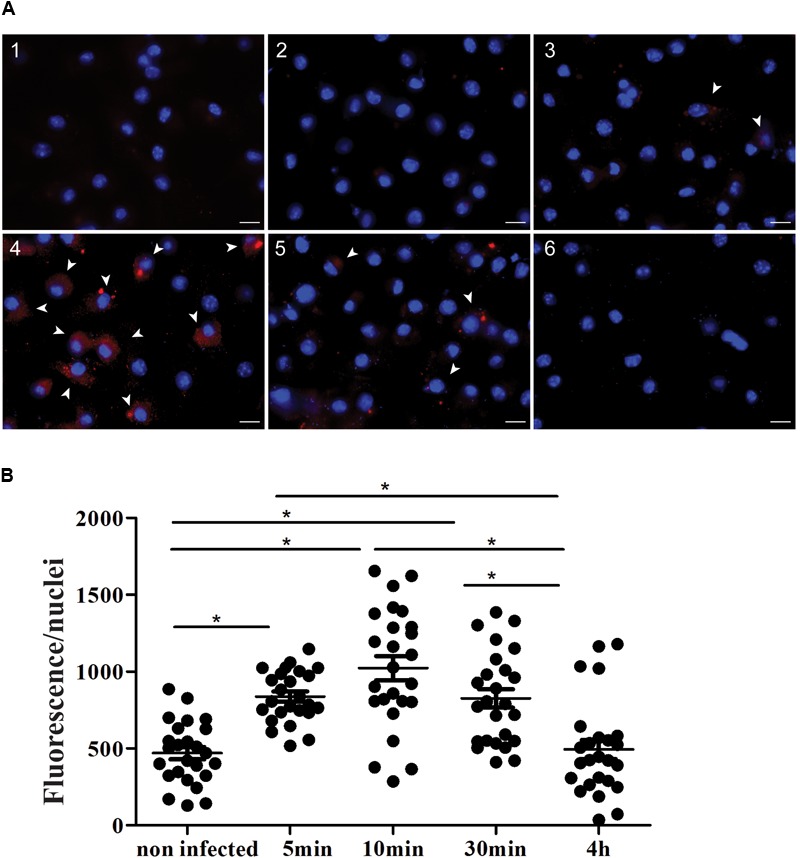
Expression of CD100 in peritoneal macrophages infected or not with promastigotes of *L. (L.) amazonensis*. **(A)** Immunofluorescence staining for CD100 on infected and non-infected macrophages. Peritoneal macrophages from BALB/c mice non-infected (1 and 2), infected with promastigotes of *L. (L.) amazonensis* at MOI of 10:1 for 5 min (3), 10 min (4), 30 min (5), and 4 h (6). Staining with anti-CD100 antibody and secondary anti-rat Alexa Fluor 568 (red) and DAPI (blue nucleus). Control: DAPI (1) and secondary anti-rat Alexa Fluor 568 + DAPI (2). Images were captured in a DMI6000B/AF6000 (Leica) fluorescence microscope coupled to a digital camera system (DFC 365 FX). White bars correspond to 10 μm. **(B)** Quantification of red fluorescence/nuclei in 25 fields for each condition. Statistical analysis by ANOVA with Tukey’s post-test, significant differences are labeled as ^∗^*p* ≤ 0.05.

Although CD100 expression in macrophages has already been documented (Kikutani and Kumanogoh, 2003; Nkyimbeng-Takwi and Chapoval, 2011), its expression and effects during parasitic infection were never explored. Thus, we have performed labeling and quantification of CD100 protein in peritoneal BALB/c macrophages under controlled conditions and at different time points following infection with *L*. (*L*.) *amazonensis* promastigotes at a MOI (multiplicity of infection) of 10 parasites per macrophage. We observed that CD100 protein levels are altered following infection in a time dependent manner (**Figure 1A**): intracellular CD100 increases between 5 and 30 min after infection and then returns to basal levels within 4 h (**Figure 1B**).

### Soluble CD100 Increases Infection of Macrophages by *Leishmania (L.) amazonensis*

After demonstrating that *Leishmania* infection increases macrophage CD100 endogenous levels, we evaluated whether the host cell infection by the parasite was affected by soluble CD100 (sCD100) added to the media. In the *Leishmania* lesion environment, sCD100 may be shed by macrophages, which express low levels of the protein (Sierra et al., 2008; Li et al., 2009; Luque et al., 2013), or by other cells, mainly activated T cells, which are known to release sCD100 (Wang et al., 2001). We thus analyzed the role of exogenous sCD100 on macrophage infection by *Leishmania*. We produced sCD100-Fc recombinant protein (from now on named as sCD100) in HEK293T and incubated macrophages with sCD100 before and together with *L. (L.) amazonensis* for different times.

**FIGURE 2:**
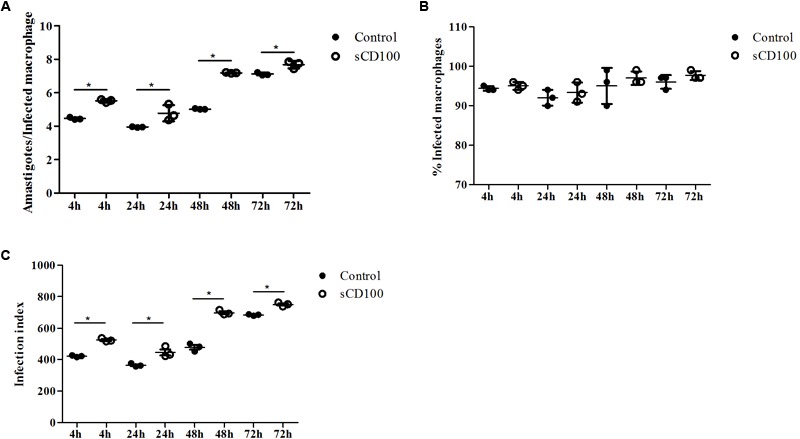
Effects of sCD100 on infection of macrophages. Macrophages from BALB/c mice were infected with *L. (L.) amazonensis* promastigotes at a MOI of 5 per 4, 24, 48, or 72 h in the presence or not of 100 ng/mL sCD100. **(A)** Amastigotes per infected cell. **(B)** Percentage of infected macrophages. **(C)** Infection index. Statistical analysis was performed by ANOVA with Tukey’s post-test, and significant differences are labeled as ^∗^*p* ≤ 0.05. Results of a representative experiment of three with similar profiles.

Our results show that the number of amastigotes per cell (**Figure 2A**), and consequently infection rates (calculated as a product of the proportion of infected cells and the number of amastigotes) (**Figure 2C**), increase significantly in the presence of sCD100 in 4, 24, 48, and 72 h of infection. On the other hand, the percentages of infected macrophages (**Figure 2B**) do not change significantly over time with or without sCD100.

### Pre-incubation of Promastigotes With sCD100 Does Not Increase Infection of Macrophages

**FIGURE 3:**
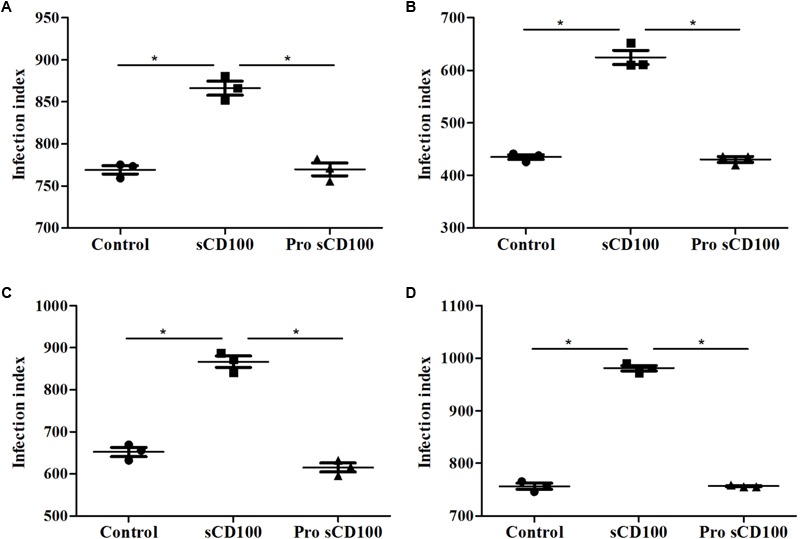
Effects of preincubation of promastigotes with sCD100 on infection index. Peritoneal macrophages from BALB/c mice were infected with *L. (L.) amazonensis* at a MOI of 5 for 4 **(A)**, 24 **(B)**, 48 **(C)**, or 72 **(D)** hours at different conditions: macrophages and parasites without sCD100 (control), promastigotes preincubated with sCD100 at a concentration of 200 ng/mL (promastigote+sCD100), macrophages and promastigotes in the continuous presence of sCD100 at a concentration of 200 ng/mL (sCD100). Statistical analysis was performed by ANOVA with Tukey’s post-test, and significant differences are labeled as ^∗^*p* ≤ 0.05. Results of a representative experiment of three.

A possible explanation for the increase of infection by *L*. (*L*.) *amazonensis* induced by sCD100 could be the binding of this molecule to the parasite, promoting its adhesion to a CD100 receptor present on the macrophage or to Fc receptors, as sCD100 protein is fused to human Fc region of IgG1 (Kumanogoh et al., 2000). To verify the first hypothesis, we pre-incubated promastigotes with sCD100 prior to macrophage infection. Again, infection was performed for 4, 24, 28, and 72 h. No significant difference in the number of macrophages infected with *L*. (*L*.) *amazonensis* or in the number of amastigote forms inside individual infected cells was observed by pre-incubation with sCD100 relative to controls at all time points (**Figure 3**). These data indicate that sCD100 does not increase infection by direct contact/interaction with the parasite. On the other hand, when macrophages are pre-incubated with sCD100, the infection rate increases significantly (**Figures 3A–D**, control vs. sCD100).

**FIGURE 4:**
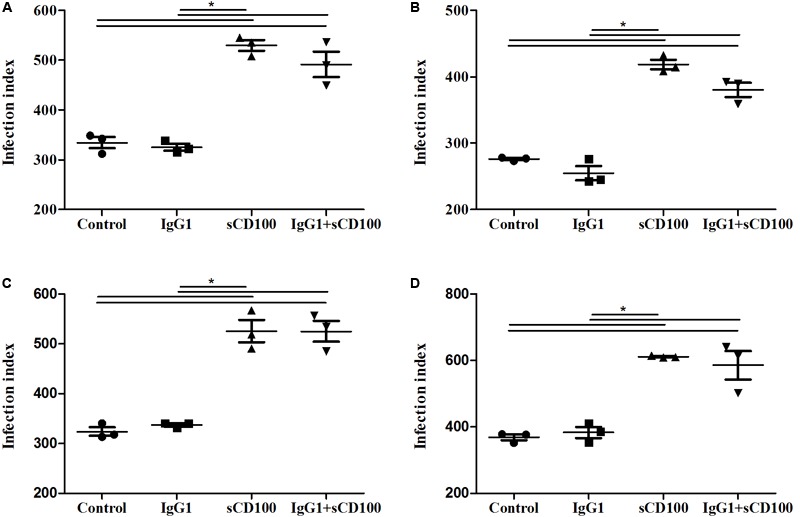
Effects of IgG1 competition on infection index. Peritoneal macrophages from BALB/c mice were infected with *L. (L.) amazonensis* at a MOI of 5 for 4 **(A)**, 24 **(B)**, 48 **(C)**, and 72 **(D)** hours in the absence of stimulus (control), in the presence of 200 ng/mL of sCD100, 70 ng/mL of human IgG1 or IgG1+sCD100. Statistical analysis was performed by ANOVA with Tukey’s post-test, and significant differences are labeled as ^∗^*p* ≤ 0.05. Results of a representative experiment of three.

Because the recombinant sCD100 that we used is produced as fusion between sCD100 and the Fc portion of IgG1, we next analyzed whether its effect on phagocytosis could be due to an interaction of the Fc IgG1 portion with the macrophage Fc receptor (FcR). To evaluate this possibility, macrophage infection assays were repeated in the presence of recombinant Fc region of human IgG1. The same control protein has been used in different studies, including one with *Leishmania* infection (Cortez et al., 2011). As expected, sCD100 alone increased macrophage infection by *L*. (*L*.) *amazonensis* while soluble IgG1 had no effect (**Figure 4**). Similar results were obtained when we blocked Fc receptor using the commercial blockers CD16 and CD32 (Supplementary Figure 1). Taken together, these results demonstrate that the increase in infection is directly mediated by sCD100.

### CD72 Is Expressed in Macrophages and Mediates sCD100 Effects on Infection

**FIGURE 5:**
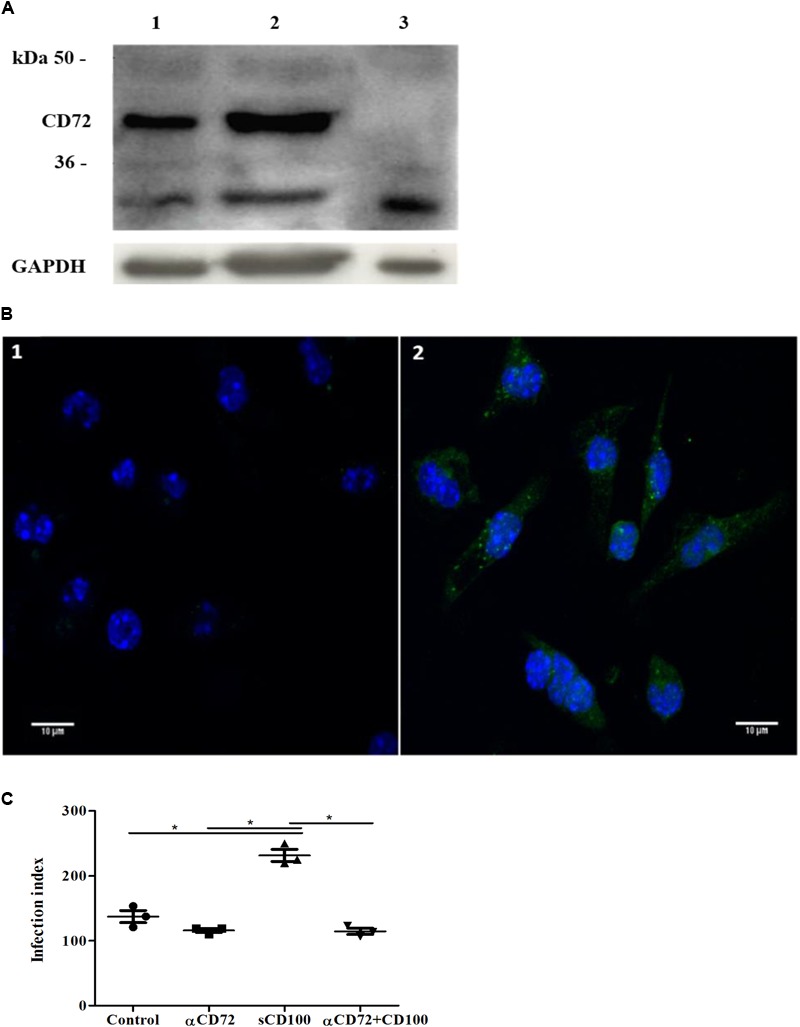
Expression of CD72 and its role in sCD100 effects on macrophages. **(A)** Western blot showing expression of CD72 and GAPDH in peritoneal macrophages from BALB/c mice (lane 1), macrophage incubated with 200 ng/mL of sCD100 for 48 h (lane 2) and L929 cells (negative control - lane 3). Thirty micrograms of protein extracts were analyzed in 10% SDS–PAGE. **(B)** Immunofluorescence staining for CD72 in peritoneal macrophages from BALB/c mice: control incubated with anti-rabbit Alexa Fluor 488 secondary antibody and DAPI (image 1), macrophages incubated with anti-CD72, anti-rabbit Alexa Fluor 488 secondary antibody and DAPI (image 2). Images were captured in Zeiss LSM 780-NLO confocal microscope, magnifying 63 ×, 1.0 zoom. **(C)** Infection index of macrophages from BALB/c mice in the presence or absence of sCD100, preincubated or not with anti-CD72 for 48 h. Data represent means and standard deviations of one experiment with triplicates. Statistical analysis was performed by ANOVA followed by Tukey’s post-test, and significant differences are labeled as ^∗^*p* ≤ 0.05.

CD72 is considered the main receptor for CD100 in macrophages (Kumanogoh et al., 2000; Ishida et al., 2003; Smith et al., 2011), but its expression has not been analyzed in murine peritoneal macrophages. We thus verified the expression of this receptor in these cells. By Western blot we observed the reactivity of CD72 immunospecific antibodies with a 45 kDa protein, the expected molecular weight for this receptor (**Figure 5A**, lane 1). Its expression is not altered upon incubation of the macrophages with sCD100, since we observed similar levels (ratios of CD72/GAPDH, the endogenous control used) in macrophages plated in the presence or not of sCD100 (**Figure 5A**, lane 2). An unrelated cell line (L929 fibroblast), which does not express CD72, was used as negative control (**Figure 5A**, lane 3). By immunofluorescence, we observed CD72 labeling in peritoneal macrophages (**Figure 5B**), confirming the presence of the receptor in these cells.

Next, we evaluated whether blockage of CD72 would affect sCD100-induced increase in macrophage infection. Macrophages were preincubated with anti-CD72 antibodies and infected with *Leishmania*. We observed that anti-CD72 antibodies alone have no effect on macrophage infection (**Figure 5C**, lane 2), as the number of infected cells is similar to control, and that sCD100 increases macrophage infection, as expected. However, when anti-CD72 antibody is added before sCD100, infection levels returned to basal levels (**Figure 5C**). These results indicate that sCD100 increases *L. (L.) amazonensis* infection in a CD72 dependent manner.

### sCD100 Increases Phagocytosis of *L.* (*L*.) *amazonensis* and Zymosan Particles

**FIGURE 6:**
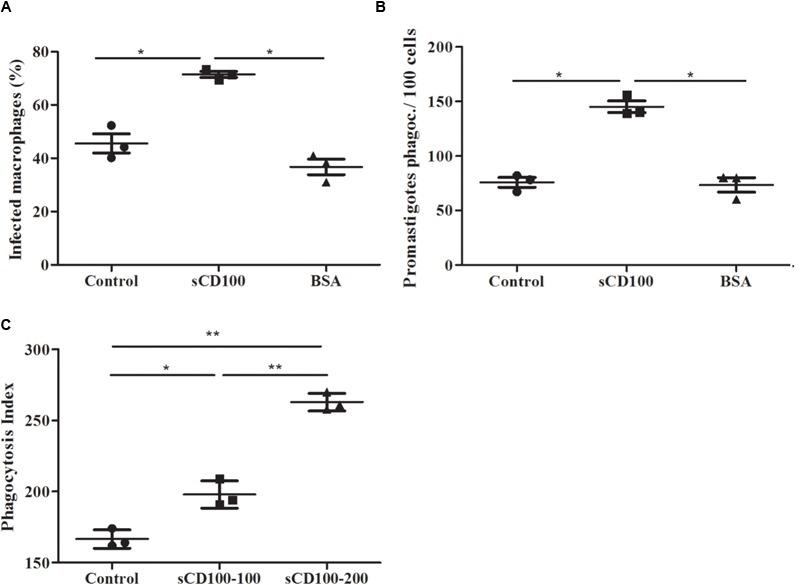
Effect of sCD100 on phagocytosis of *Leishmania* and zymosan by peritoneal macrophages from BALB/c. **(A)** Percentage of infected macrophages and **(B)** promastigotes phagocytosed by 100 macrophages in the continuous presence of 100 ng/mL sCD100 or BSA or with no stimulus (control), analyzed by immunofluorescence. Data represent means and standard deviations of three independent experiments. **(C)** Phagocytosis index of zymosan using 1 particle per macrophage for 1 h in the continuous presence of 100 or 200 ng/mL sCD100 or with no stimulus (control). Results of a representative experiment out of three with similar values. Statistical analysis was performed by ANOVA followed by Tukey’s post-test, and significant differences are labeled as ^∗^*p* ≤ 0.05.

We have shown that sCD100 increases infection of macrophages after 4 h of incubation with *Leishmania*, suggesting that the molecule could participate in the initial steps of phagocytosis. To test this hypothesis, we performed a phagocytosis assay in which macrophages were plated (overnight) in the presence or absence of sCD100 and then incubated with promastigotes in the presence or abscence of sCD100 for only 5 min. Promastigotes attached to the host cell were visualized by fluorescent microscopy in green and blue (anti-*Leishmania* and DAPI, respectively), while internalized parasites were labeled only in blue (DAPI) (Supplementary Figure 2). Phagocytosis of *Leishmania* was assessed quantitatively by the percentage of infected macrophages and the total number of promastigotes phagocytosed. Phagocytosis of zymosan was also analyzed. An increase in infected macrophages and internalized promastigotes (**Figures 6A,B**) was observed in the presence of sCD100, indicating that it affects the initial steps of phagocytosis. Besides, there was a significant increase in the phagocytosis of zymosan (represented by the phagocytosis index) at both sCD100 concentrations tested (**Figure 6C**), indicating that this protein augments phagocytosis in general and not specifically of *Leishmania* parasites.

### sCD100 Does Not Affect Actin Polymerization

**FIGURE 7:**
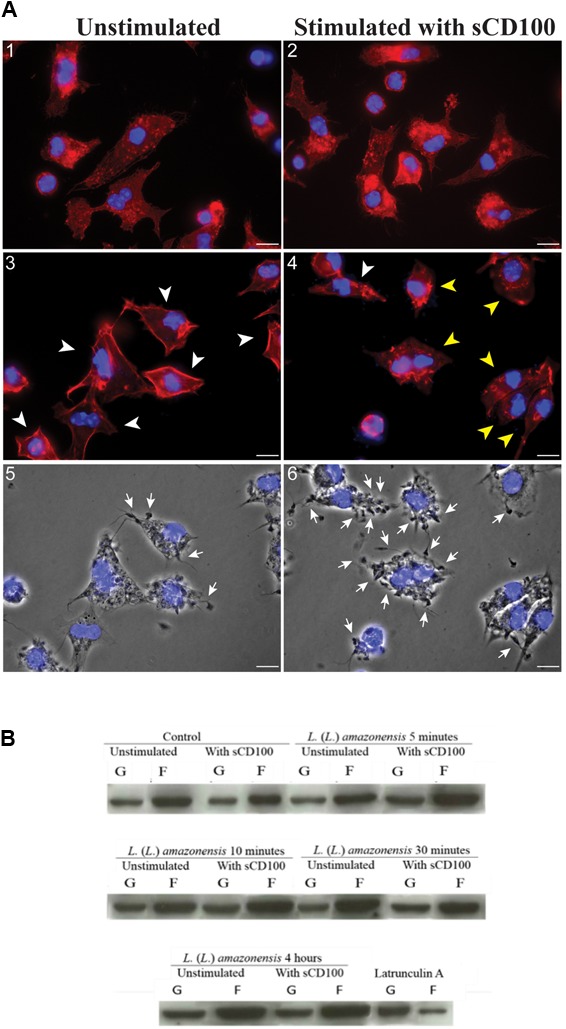
Effect of sCD100 on actin polymerization in infected and non-infected macrophages. **(A)** Peritoneal macrophages from BALB/c mice unstimulated (1, 3, 5) or stimulated with 200 ng/mL of sCD100 (2, 4, 6) were infected with *L. (L.) amazonensis* at MOI of 10 for 30 min (1, 2) or 4 h (3, 4), followed by staining with phalloidin Red: Actin (labeled with phalloidin-Texas Red), Blue: nuclear DNA and kinetoplast (DAPI). Images were captured in DMI6000B/AF6000 (Leica) fluorescence microscope coupled to a digital camera system (DFC 365 FX). White bars correspond to 10 μm. The arrowheads point to macrophages presenting cortical (white) or cytoplasmic (yellow) organization of F-actin. Contrast phase of the samples at 4 h from unstimulated and stimulated with sCD100 (5, 6). The arrows point to parasites attached to macrophages. The images are representative of two independent experiments. **(B)** Western blot for actin in soluble and insoluble fractions of bone marrow macrophages incubated with or without sCD100, infected or not for 5, 10, 30 min or 4 h. This representative image shows soluble actin (supernatant, G-actin), and polymerized actin (pellet, F-actin) bands, and the graph shows F/G actin ratio after densitometry. Results of a representative experiment of three with the same profile.

Since *Leishmania* is internalized in an actin-dependent process, we analyzed whether sCD100 modulated actin polymerization. Actin was analyzed in macrophages plated in the presence or abscence of sCD100 and infected with promastigotes in the presence or abscence of sCD100 for different periods. **Figure 7A** shows representative confocal images of immunofluorescence of macrophages incubated with sCD100 and infected for 30 min and 4 h, as well as the same conditions with no sCD100. The analysis of multiple fields at 4 h of infection showed that F-actin (polymerized actin, labeled with Texas Red Phalloidin) has a different organization in macrophages stimulated with sCD100. In fact, while a cortical distribution (arrowhead) of F-actin is shown in the absence of sCD100 (**Figure 7A**, image 3), the presence of sCD100 leads to a more heterogeneous and cytoplasmic distribution of polymerized actin (**Figure 7A**, image 4). A cytoplasmic pattern of F-actin was also visualized at 30 min of infection, in both conditions (**Figure 7A**, images 1, 2). Confirming our previous results, the presence of sCD100 at 4 h of infection increased the number of promastigotes attached (arrows) and internalized by macrophages (**Figure 7A**, images 6,5, respectively), suggesting a correlation between the effect of sCD100 on F-actin dynamics and *Leishmania* infection.

Since small differences in F-actin polymerization may not be perceived by immunofluorescence, we evaluated actin polymerization in control macrophages and in macrophages treated with sCD100, infected or not with *L. (L.) amazonensis* by Western blot (**Figure 7B**). The experiment was based on the separation of F-actin (insoluble) and G-actin (soluble) by ultracentrifugation, followed by blotting for actin in the two fractions. As a technical control, we employed extracts of macrophages treated with Latrunculin A, which blocks actin polymerization (Oliveira et al., 1996). Under all conditions F-actin band is about two times more intense than G-actin band, irrespective of sCD100 stimulus or infection by *L.* (*L*.) *amazonensis* (**Figure 7B**, representative experiment). Thus, no clear correlation between sCD100 and actin polymerization can be drawn. The control with Latrunculin A shows a clear decrease in the F-actin band, indicating that the separation of soluble and insoluble actin was efficient using this method.

### Soluble CD100 Increases Infection of Macrophages by *Trypanosoma cruzi*

We have here shown that sCD100 binds to macrophage CD72 and increases *Leishmania* phagocytosis. We have also shown that treatment of macrophages with sCD100 increases phagocytosis of zymosan, indicating that this effect is not specific for *Leishmania*. Therefore, we decided to investigate whether cCD100 could modulate infection of macrophage by other parasites.

**FIGURE 8:**
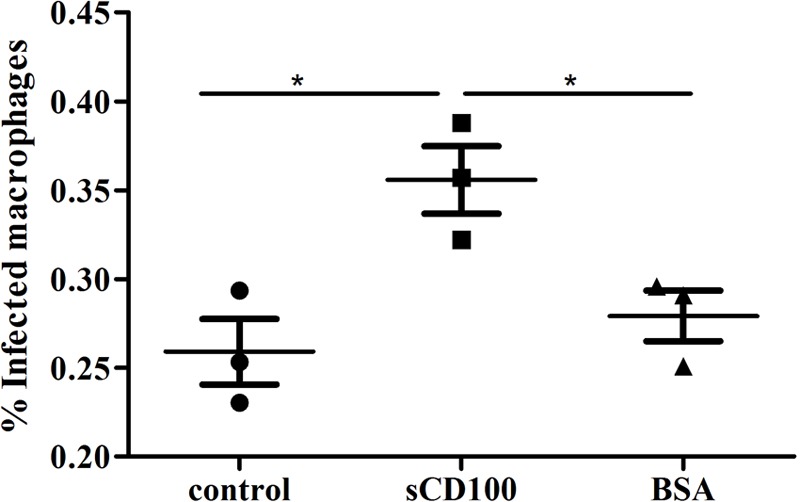
Effects of sCD100 on infection of macrophages by Trypanosoma cruzi. Macrophages from BALB/c mice were infected with *T. cruzi* trypomastigotes at a MOI of 10 for 24 h in the presence or not of 200 ng/mL sCD100. Statistical analysis was performed by ANOVA with Tukey’s post-test, and significant differences are labeled as ^∗^*p* ≤ 0.05. Results of one experiment representative of two with similar profile.

*Trypanosoma cruzi* has several developmental stages, and the infective trypomastigotes can enter almost all nucleated cells of the vertebrate host, phagocytic or non-phagocytic. Tissue resident macrophages are importante targets for early infection, and parasite entry into these cells can occur by a phagocytic-like or a non-phagocytic mechanism (Epting et al., 2010; Barrias et al., 2013). We thus analyzed whether sCD100 could also affect macrophage infection by *T. cruzi* trypomastigotes. Trypomastigotes were incubated with macrophages in the presence or absence of sCD100 and the percentage of infected cells was quantified. Similar to *Leishmania*, we observed that sCD100 promoted a significant increase in cell infection by *T. cruzi* (**Figure 8**). These results suggest that CD100 may play an important role in macrophage infection by different trypanosomatid parasites.

## Discussion

Monocytes and the mature macrophage cells are key elements in immunity, and their role in parasitic infection has been clearly demonstrated for trypanosomatids (Dos-Santos et al., 2016). Understanding the molecular mechanisms of parasite phagocytosis by these cells is an important step toward the development of novel therapeutic options for infected patients, in particular for visceral leishmaniasis, for which treatment is necessary but therapeutic options are usually toxic and with poor efficacy (McGwire and Satoskar, 2014). CD100 is an important molecule involved in the communication between immune cells (Kumanogoh et al., 2000; Shi et al., 2000; Mizui et al., 2009), but its role in macrophage functions has been poorly studied. In fact, the only report concerning CD100 and phagocytosis by these cells is a previous paper from our group showing that soluble CD100 (sCD100) decreases internalization of oxidized LDL by human macrophages by inhibiting the expression of the scavenger receptor CD36 (Luque et al., 2015).

Apart from their role in immune response, macrophages are the main host cells for *Leishmania*. We here showed that these cells respond to sCD100 by increasing phagocytosis of *Leishmania, Trypanosoma cruzi* and zymosan. Zymosan is a β-glucan rich particle derived from the yeast *Saccharomyces cerevisiae*, frequently used as a model for phagocytosis. Non-opsonized zymosan is mainly recognized by macrophage dectin-1 receptor (Brown et al., 2002), which is not involved in *Leishmania* phagocytosis. *T. cruzi* is internalized by host cells via multiple endocytic pathways. Entry into macrophages occurs mainly but not only by phagocytosis (Epting et al., 2010; Barrias et al., 2013), and involves several macrophage receptors, some of them different from the ones used for *Leishmania* internalization. Thus, the effect of sCD100 on phagocytosis probably does not depend on a specific phagocytic receptor of macrophages.

The increase in *Leishmania* infection promoted by sCD100 depends on its interaction with CD72 receptor. The effects of sCD100 on phagocytosis may differ in murine and human macrophages. In fact, CD72 is the main CD100 receptor in murine macrophages (Tamagnone et al., 1999; Kumanogoh et al., 2000), while we have shown that plexin B2 is an effective CD100 receptor in human monocytes and macrophages (Luque et al., 2015). CD72 was shown to be expressed in bronchial epithelial cells, alveolar macrophages, B cells, dendritic cells, fibroblasts, basophils (Kikutani and Kumanogoh, 2003; Mizui et al., 2009; Smith et al., 2011), and we here show its expression also in murine resident peritoneal macrophages. CD72 belongs to the superfamily of C-type lectins, which contain a cytoplasmic domain with two tyrosine inhibitory motifs (ITIMs) that when phosphorylated bind to the protein tyrosine phosphatase 1 (SHP-1) and the adapter protein Grb2 (Wu and Bondada, 2009; Nkyimbeng-Takwi and Chapoval, 2011). No study has ever analyzed CD72 signaling in macrophages, but most studies show that binding of CD100 to CD72 in B cell reverse the inhibitory potential of CD72 to cause dephosphorylation ITIM and release of SHP-1 (Wu and Bondada, 2009). It will be interesting to analyze whether sCD100 binding to macrophage affects not only parasite entry, as we demonstrated here, but also further signaling events such as ITIM phosphorylation and SHP1 release from the CD72 cytoplasmic tail. Infection in the presence of sCD100 led to changes in F-actin organization. Indeed, the cortical distribution of F-actin observed in macrophages at 4 h of infection changed to a heterogeneous and cytoplasmic pattern in the presence of sCD100. Similar changes in actin organization have already been reported in medullary macrophages when infected with *Leishmania amazonensis* (de Menezes et al., 2017) and in cultured chromaffin cells compared to cells in adrenomedullary tissue (Gimenez-Molina et al., 2017).

This work is the first to report the role of CD100 in parasitic infection. Further studies must be performed to unravel how CD100 binding to macrophage CD72 increases phagocytosis of zymosan, *Leishmania* and *T. cruzi*, which interact with different surface receptors. Further studies should also address the role of sCD100 in *in vivo* models of leishmaniasis, and on *Leishmania* phagocytosis by human macrophages.

## Author Contributions

MG was the student responsible for the project, who performed most experiments, wrote the paper, and prepared most figures. ER performed immunofluorescence for CD100, prepared the corresponding figure, and revised the paper. FF helped in experimental design and phosphatase experiment (not included) and revised the paper. MC contributed with actin separation and immunofluorescence experiments and revised the paper. MCC performed confocal microscopy. AT and RG designed and performed *T. cruzi* infection experiments and revised the paper. BS designed and supervised the project, wrote the paper, and was responsible for the funding.

## Conflict of Interest Statement

The authors declare that the research was conducted in the absence of any commercial or financial relationships that could be construed as a potential conflict of interest.
