# Supplementary material for: CD100/Sema4D Increases Macrophage Infection by Leishmania (Leishmania) amazonensis in a CD72 Dependent Manner
Source: Front Microbiol. 2018 Jun 5;9:1177. doi: 10.3389/fmicb.2018.01177 (PMC5996280; doi:10.3389/fmicb.2018.01177)
Supplement: Supplementary file 1 [file Data_Sheet_1.docx]

Supplementary Material

CD100/Sema4D increases macrophage infection by *Leishmania (Leishmania) amazonensis* in a CD72 dependent manner

Mariana K. Galuppo , Eloiza de Rezende, Fabio Luis Forti, Mauro Cortez, Mario Cruz, Andre Teixeira, Ricardo J. Giordano, Beatriz S. Stolf*

*Corresponding author: [bstolf@usp.br](mailto:bstolf@usp.br)

**
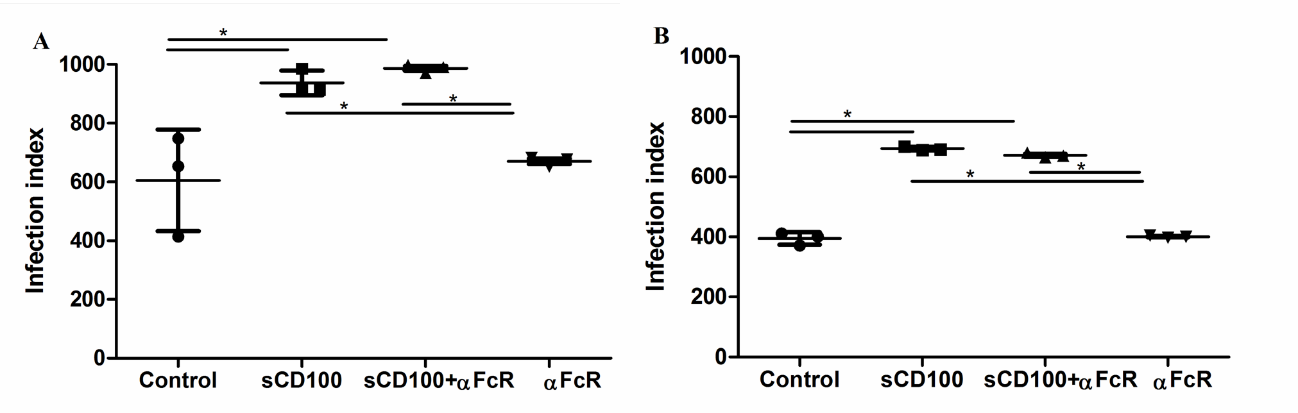
**

**Supplementary figure 1**. Infection index of peritoneal macrophages with *L*. (*L*.) *amazonensis* for 4 (A) and 24 (B) hours in the continuous presence of sCD100 (200 ng/ mL), sCD100 + FcR blocker (0.01 μg / mL) and FcR blocker. Mean and deviation of an experiment with triplicates. Statistical analysis used: ANOVA. *: P≤0.05.

**
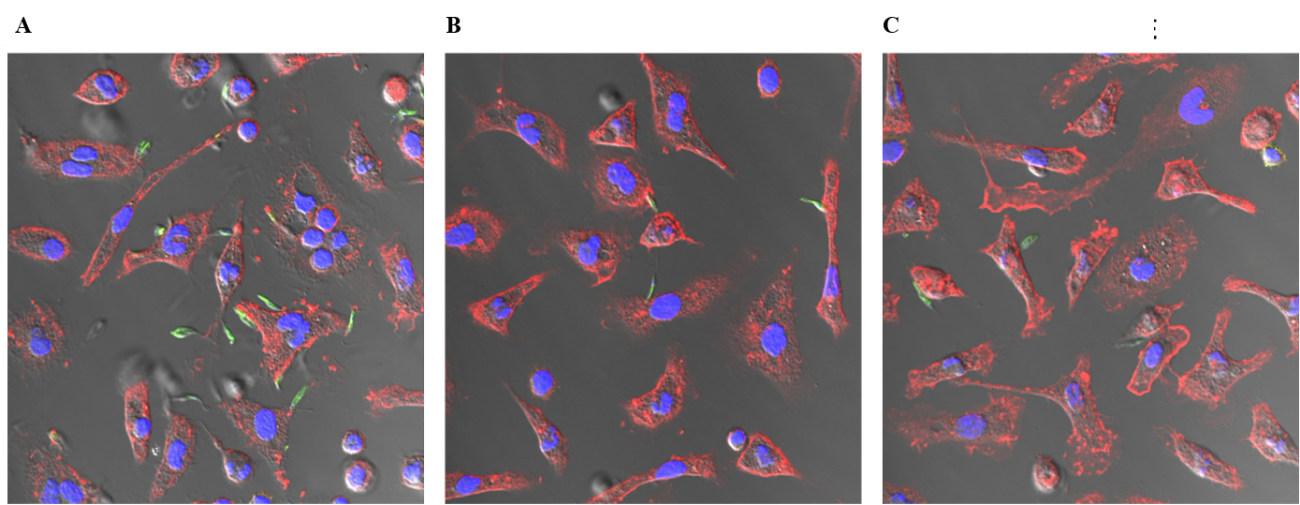
**

**Supplementary figure 2**. Immunofluorescence of phagocytosis of *L*. (*L*.) *amazonensis* promastigotes by peritoneal macrophages of BALB/c mice. Green: *Leishmania* (primary anti-*Leishmania*, secondary anti mouse Alexa fluor 488), Red: actin (labeling with phalloidin), Blue: nuclear DNA and cynetoplast (DAPI). **A.** Macrophage stimulated with sCD100. **B.** Macrophage stimulated with BSA. **C.** Unstimulated macrophage. Confocal Microscope, 63x magnification, 1.0 zoom.
